# Tidal variability of water quality parameters in a mesotidal estuary (Sado Estuary, Portugal)

**DOI:** 10.1038/s41598-021-02603-6

**Published:** 2021-11-30

**Authors:** Ângela Nascimento, Beatriz Biguino, Carlos Borges, Rui Cereja, Joana P. C. Cruz, Fátima Sousa, Joaquim Dias, Vanda Brotas, Carla Palma, Ana C. Brito

**Affiliations:** 1 MARE - Marine and Environmental Sciences Centre, Faculdade de Ciências, Universidade de Lisboa, 1749-016 Lisboa, Portugal; 2 Instituto Hidrográfico, Rua das Trinas 49, 1249-093 Lisboa, Portugal; 3 Faculdade de Ciências, IDL - Instituto Dom Luiz, Universidade de Lisboa, 1749-016 Lisboa, Portugal; 4 Departamento de Engenharia Geográfica, Geofísica e Energia (DEGGE), Faculdade de Ciências, Universidade de Lisboa, 1749-016 Lisboa, Portugal; 5 Departamento de Biologia Vegetal, Faculdade de Ciências, Universidade de Lisboa, 1749-016 Lisboa, Portugal

**Keywords:** Environmental sciences, Ocean sciences

## Abstract

To establish effective water quality monitoring strategies in estuaries, it is imperative to identify and understand the main drivers for the variation of water quality parameters. The tidal effect is an important factor of the daily and fortnightly variability in several estuaries. However, the extent of that influence on the different physicochemical and biological parameters is still overlooked in some estuarine systems, such as the Sado Estuary, a mesotidal estuary located on the west coast of Portugal. The main objective of this study was to determine how the water quality parameters of the Sado Estuary varied with the fortnightly and the semidiurnal tidal variation. To achieve this goal, sampling campaigns were conducted in May/18, Nov/18 and Jun/19, under neap and spring tidal conditions, with data collection over the tidal cycle. Results were observed to be significantly influenced by the tidal variation, in a large area of the estuary. Flood seemed to mitigate possible effects of nutrient enrichment in the water column. Additionally, significant differences were also observed when considering the different sampling stations. Temperature, Suspended Particulate Matter (SPM) and nutrients showed the highest values at low water. Lastly, the implications of the tidal variability in the evaluation of the water quality according to Water Framework Directive were also discussed, highlighting the importance of studying short-time scale variations and the worst-case scenario to ensure water quality is maintained. These findings are relevant for the implementation of regional management plans and to promote sustainable development.

## Introduction

Estuaries are transitional areas, encompassing freshwater and marine ecosystems. They are recognized as highly productive areas, being important habitats for local wildlife and protection areas for many species, mainly due to their calm waters. For all these particularities, estuaries were sought by mankind from an early age. Currently, they represent the regions where the most populous cities on the planet are located^1^. They constitute popular areas for recreation, transport and commercial activities that, combined with the high aesthetic value characteristic of this type of environments, raise the importance of the region. Estuaries are also vital waterways that play a key role in the economy and the environment^2^. However, worldwide, a decline in the health of estuaries is being observed due to this continuous and intensive anthropogenic presence^3,4^.

Naturally, estuaries are very dynamic systems, where a high temporal and spatial variability of the physicochemical and biological parameters is easily detected. There are several factors that can enhance this variability, however, the extent of their influence in the estuary dynamics may be difficult to quantify. This variability is primarily induced by the mixture of fresh river discharges with salty oceanic water^5^. The river flow is one of the main factors responsible for the transport of sediments and nutrients^6^, as well as the land runoff, and tends to influence the innermost regions of estuaries more than its outermost areas^7^. The increase in nutrients may lead to an increase in phytoplankton biomass, and consequently, to higher concentrations of chlorophyll *a* (*e.g*., Howarth and Marino^8^; Bricker et al.^3^; Nixon^9^). However, the river flow is subject to seasonal variations of the weather conditions, being higher with intense rainfall (as is also observed with land runoff). This means that the area of the estuary most affected by the river outflow may vary, as well as the spatial and temporal distributions of the parameters directly dependent on the fluvial variation. The same is observed with the presence of oceanic water entering through the mouth of estuaries, usually presenting higher salinity and pH^10^. As a consequence, salinity and pH are usually higher near the estuary mouth. In the particular case of the Sado Estuary, an urbanized estuary on the west coast of Portugal, the spatial and seasonal variability of the physicochemical and biological parameters of the water has been recently studied using satellite remote sensing data^11^. According to these authors, the Sado Estuary should not be considered spatially homogeneous, as the innermost region of the estuary shows higher values of chlorophyll *a*, suspended particulate matter (SPM), colored dissolved organic matter and turbidity throughout the year. This region is also the most susceptible to seasonal variations of the weather and along the estuary, these parameters showed higher values during spring and summer^11^. As for the nutrients, they are usually detected in higher concentrations in the inner channels of the estuary^12^, similarly to what was observed in the Tagus Estuary^13^. Overall, the water quality parameters in estuaries like Sado, present a strong seasonality (mainly due the variation of air temperature and rainfall) and vary in response to physical transport processes^14^, varying with the river flow and the tidal cycle.

Worldwide, analyses focusing on the influence of the tide, especially the daily cycles, in the variability of the physicochemical and biological parameters in estuaries are still missing. Fortune and Mauraud^15^ concluded that the water quality of Jones Creek in Darwin Harbour (Australia) was influenced by the fortnightly tidal cycle, with spring tides typically inducing larger fluctuations in water quality. In fact, Tufoni^16^ concluded that chlorophyll *a* and turbidity were highly variable at fortnightly, being higher during spring tidal conditions, like the dissolved oxygen and pH (inner Ria Formosa, Portugal). On the other hand, in Pages and Futch Creeks (North Carolina, USA), the most significant variability among most parameters was the daily tidal variation^17^. Correia et al.^18^ studied the effect of the tide in the Arade Estuary (Portugal) and concluded that the salinity, pH and oxygen, varied, in general, in phase with the height of the tide, which was also reported by Das et al.^19^ for the Bay of Bengal. In opposition, Correia et al.^18^ discussed how nutrients, chlorophyll *a* and suspended solids, varied in antiphase with the height of the tide. However, these works also referred that the simultaneous influence of solar radiation input on the variation of dissolved oxygen (and possibly the temperature) may act as an obstacle to the analysis. Still, Wetz et al.^20^ also detected a decrease of chlorophyll *a* from low tide to 1 h after high tide, by about 47 to 51%, in the creeks of North Inlet (South Carolina, USA). For the Tagus Estuary, Cereja et al.^13^ indicated that the fortnightly spring-neap tidal cycle presented a significant influence on the variation of the chlorophyll *a* concentration, with high frequency phenomena explaining a relevant part of its variability (up to 50%), while seasonality was observed to explain only ≈ 35% of its variability. In this estuary, neap tide enhances higher temperatures, NO_3_^−^ and NH_4_^+^^21^. Moreover, Cereja et al.^13^ suggested that low water induced the highest concentrations of SPM and nutrients in specific areas of the estuary. Although some studies exist, it is important to note that estuaries are characterized as much by similarities as by differences^22^, thus is key to conduct site-specific analysis of the tidal effect on water quality parameters.

Given that the Sado Estuary is a well-mixed estuary whose circulation is mainly dominated by the tidal action^23,24^, it is possible to assume the tidal variation as one of the main drivers of the water quality variation. Quantifying the influence of the tides in the variation of the physicochemical and biological parameters in an estuary is a key contribution to understand its dynamics, to implement effective monitoring plans that take into account the worst-case conditions and to guarantee good water quality status^25^. With the purpose to increase the performance of water quality analysis and standardizing their methodologies, the European Union published the Water Framework Directive (WFD) (2000/60/EC)^26^. The WFD states that all Member States need to achieve and/or maintain "good status" for all water bodies, including estuaries (transitional water bodies). To comply with the requirements of the WFD and achieve the proposed objectives, it is imperative to conduct regular monitoring operations. In Portugal, the WFD has already encouraged several estuarine studies, such as the work of Brito et al.^27^, which defined phytoplankton class boundaries considering several Portuguese estuaries, or Caetano et al.^28^, whose analysis was focused in defining benchmark values for nutrients.

Systematic and cautious monitoring can identify water quality problems and find proposals for resolutions along decisions-makers^29^. The detection of patterns, which are only observable at the reduced time scale of the tidal cycle, can be hindered^14^ if the tidal variability of the water quality in estuaries is not considered. These reduced time scale analyses are important, in particular, for (i) aquaculture purposes, where it is important to take advantage of the variability of water parameters to ensure the best environmental conditions for production; and for (ii) satellite remote sensing studies, where sensors collect data at a fixed time of the day, thus acquiring information under different tidal conditions.

Understanding the tidal effect in the Sado Estuary is key to bridge knowledge gaps that exist on the structure and functioning of estuaries and also to improve the water quality monitoring programs in complex and urbanized mesotidal systems. Several important scientific questions are raised. How does the tidal variation affect the water quality parameters? Is the estuary acting as a source or a sink of these parameters (*e.g*., nutrients)? What is the best tidal condition to perform water quality monitoring? How far off from reality will water quality assessments be if tidal stage is not accounted for in sampling? Can we optimize monitoring programs to ensure cost-effective procedures? To address these questions, sampling was conducted in the Sado Estuary with the objective to evaluate the variability of the several physicochemical and biological parameters at the fortnightly spring-neap time scale (referred as fortnightly tidal cycle) and along the semidiurnal tidal cycle. One of the main aims of this work was also to understand the implications of the tidal variability in assessing the water quality of the Sado Estuary under the WFD and to propose recommendations to implement effective monitoring strategies.

## Materials and methods

### Sado estuary

**Figure 1:**
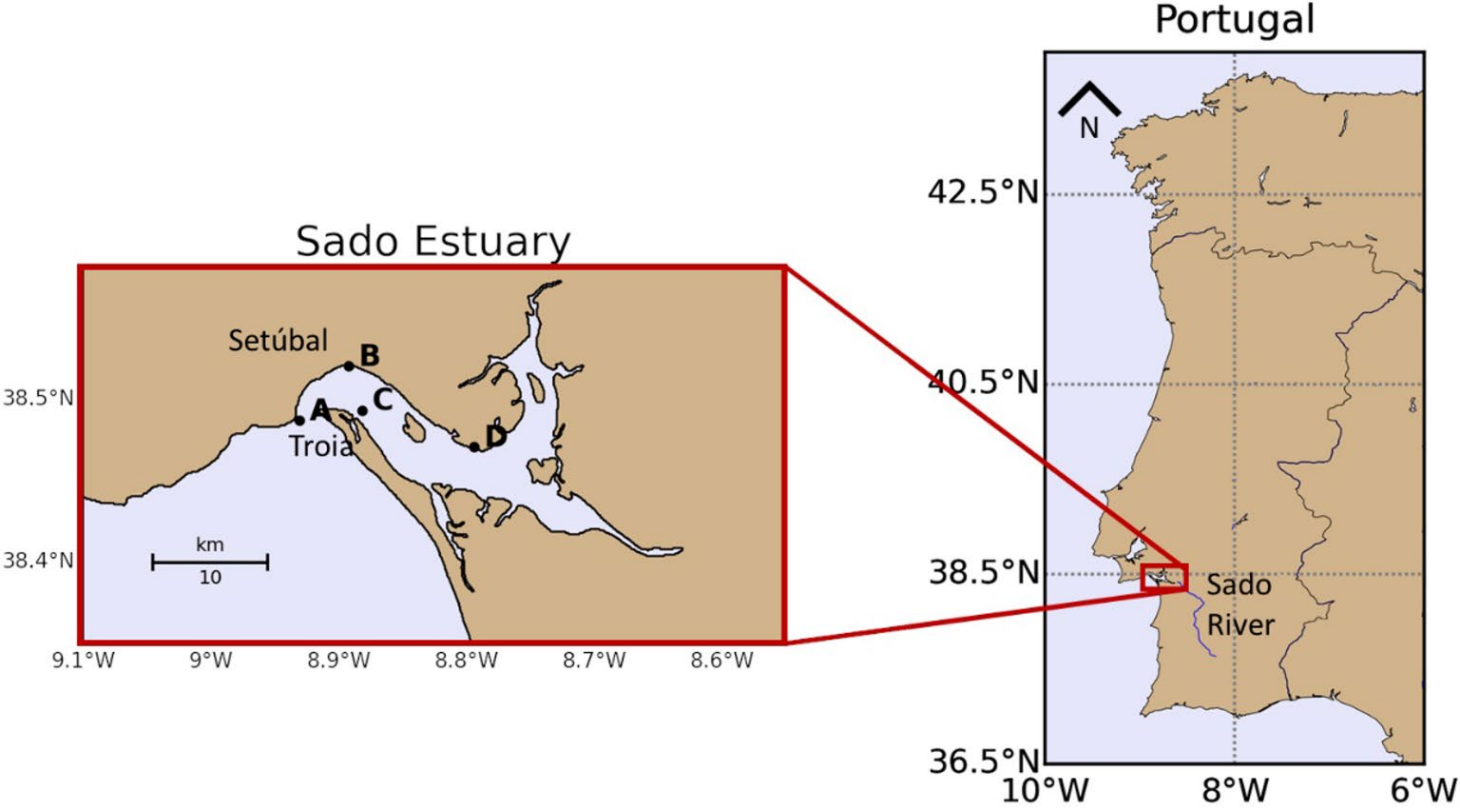
Representation of the Sado Estuary and the sampling stations A, B, C and D. Sado River course is represented in blue along the Portuguese territory. The maps were produced using Basemap package (1.2.2) from Python 3.8.

The Sado River begins in the region of Serra da Vigia and travels a course of ≈ 175 km in a southeast-northwest orientation, until it reaches the bay of Setúbal, where it meets the Atlantic Ocean^30^. This transitional zone is known as the Sado Estuary (Fig. 1) and it has a total area of 212.4 km^2^^31^. The average depth of the Sado Estuary is ≈ 10 m, but it can reach 40 m at its mouth^32^. The estuary is divided in two main navigation routes separated by intertidal shoals, the north channel and the south channel. Its innermost area includes a vast intertidal zone.

The Sado Estuary is a mesotidal estuary with semidiurnal tides^33^. The tides are the main enhancer of the circulation in the estuary^23^. The water inflow and outflow are observed through the two navigation channels, unidirectionally along the water column and according to the tidal regime. Regarding the temperature and salinity variability, Biguino et al.^24^ investigated the structure of the water column in the Sado Estuary, considering several stations with depths between 8 and 40 m, along different seasons of the year. The average temperature and salinity profiles obtained revealed well‐mixed waters, with very little stratification and a relatively high vertical homogeneity^24^. Therefore, surface water samples could be representative of the entire water column. The tidal height in the estuary goes up to 3.9 m at high water^34^.

The Sado River has a naturally low flow, mainly because the river spring is located in an arid region and the river does not experiences relevant differences of altitude in its course until reaching the mouth. Since 1970 to 2019, the daily average of the river flow was 5.6 m^3^/s^35^. Although the river flow is low, its seasonality is evident, and it is associated with the weather conditions of the region. The climate in Sado´s hydrographic basin (area of 7692 km^2^)^36^ is dry, sub-humid Mediterranean type, having a humid period that usually covers the months October–March^37^. During practically the entire sampling period, May/18 to Jun/19, the Sado hydrographic basin was under the effect of meteorological drought^38^.

Part of the estuary is recognized as a protected area since 1980, and the natural reserve has a navigable area of about 23,160 ha, including ≈ 28 km of the river course^30^. The same channels that are important water exchange routes for the circulation in the estuary, are also relevant to access the port of Setúbal, one of the most important harbors of Portugal. In addition to its economic relevance (also highlighted with the presence of chemical and cellulose industries in Setúbal area), the estuary is known for being an area of intensive agriculture land use (mainly rice fields) and for bivalve aquaculture, whilst integrating urban centers with a high population density (Setúbal, ≈ 120 × 10^3^ inhabitants^39^). Therefore, the estuary, in addition to its well-known aesthetic value and unique biodiversity, it is also marked by a significant anthropogenic influence.

### Sampling strategy

The present study focuses on the analysis of several water quality parameters measured during whole semidiurnal tidal cycles: temperature (T), practical salinity (S), pH, dissolved oxygen (DO), suspended particulate matter (SPM), chlorophyll *a* concentration, and dissolved nutrients—nitrite (NO_2_^−^), nitrate (NO_3_^−^), ammonium (NH_4_^+^), phosphate (PO_4_^3−^) and silicate (Si(OH)_4_). The total oxidized nitrogen (NO_x_) represents the sum of the nitrite and the nitrate present in a water sample (NO_x_ = NO_2_^−^ + NO_3_^−^). Also, the dissolved inorganic nitrogen (DIN) is presented as the sum of NO_2_^−^, NO_3_^−^ and NH_4_^+^ (DIN = NO_2_^−^ + NO_3_^−^ + NH_4_^+^). Lastly, the N/P ratio was used as an indicator of nutrient limitation of phytoplankton growth through the Redfield ratio, that describes the average composition of phytoplankton biomass as N:P = 16:1^40^. This ratio has been adopted as an indicator threshold for a possible nutrient limitation^41^. According to this relation, phytoplankton may be N-limited at N:P < 16 and P-limited at N:P > 16^42^.

Sampling was conducted at surface in four stations distributed along the estuary (Fig. 1). The designation and geographical coordinates of the sampling stations, and their respective depths, can be found in Table S1 of the Supplementary Materials. Six full tidal-cycle campaigns were performed in 8 and 15 May/18, 8 and 16 Nov/18, and 18 and 26 Jun/19. Two campaigns were always conducted in each month indicated above, to include neap (NT) and spring tides (ST) conditions. For tidal range and mean daily river flow information regarding each sampling campaign, please see Table S3 from Supplementary Materials.

During these campaigns, sampling was performed from sunrise to sunset due to navigation constraints. In each semidiurnal tidal cycle, sampling was conducted on cycles of approximately two hours at each station, using two boats: the first one performed sampling on stations A and C and the second one on stations D and B. In all the campaigns, a multiparameter sonde was used and water samples were collected for physicochemical and biological measurements. Tidal information was obtained from the Tidal Tables of Instituto Hidrográfico (Lisbon, Portugal).

#### Multiparameter Sonde data collection

Two multiparameter sondes (Hydrolab DS4a and DS5X, from OTT Hydromet) were used for in situ measurements of temperature and dissolved oxygen. Temperature was measured with a thermistor of sintered metallic oxide^43^. The dissolved oxygen was preferentially measured by the electrochemical method using an oxygen permeable membrane^44^. For quality control of in situ DO measurements, a confirmation method was used: the Winkler method^45^ adapted to saline waters or a polarographic sonde.

#### Water samples collection and preservation

At every station, water samples were collected at the surface (< 1 m depth) using 8 L Niskin bottles. These samples were obtained to quantify salinity, pH, DO, SPM, chlorophyll *a* and dissolved nutrients.

As the water samples required to be quickly filtered and preserved or promptly analyzed, a field laboratory was set up at the facilities of the Captaincy of the Port of Setúbal. On the sampling day, water samples were filtered using: (i) for SPM, previously washed and weighed type GF/B glass fiber filters of 1.0 μm nominal pore size; (ii) for chlorophyll *a*, cellulose ester filters of 0.45 μm porosity; (iii) for salinity and dissolved nutrients, polycarbonate filters with 0.40 μm porosity. Also, on the sampling day, pH and DO analysis by iodometry were performed in the field laboratory as described in the next section.

The filters for SPM were kept in bottles in the refrigerator for a maximum of 7 days and the filters for the quantification of chlorophyll *a* were frozen at − 20 °C, to be analyzed later on. To quantify the salinity and the nutrients, the water samples were filtered and kept in bottles: the bottles to determine the salinity were kept at room temperature and the bottles for quantifying nutrients were frozen at  − 20 °C until analysis.

### Processing in the laboratory

Salinity was measured using a Guildline Autosal 8400B high precision salinometer^46,47^. The pH was quantified using a calibrated Metrohm benchtop 744 pH meter, with a combined glass membrane electrode and a built-in Pt1000 temperature sensor (adapted from SMEWW^43^. Regarding SPM quantification, upon filtration and removal of salts by flushing the SPM filters with an adequate volume of deionized water, these were placed in an oven at 105 ºC until total removal of water. After being in the oven, they went to a desiccator to reach room temperature. Then, the filters were weighed and the difference in weight recorded between the original filter weight and the sample filter weight, affected by the volume of sample filtered, corresponded to the amount of total suspended matter existing in the water sample^48^. Chlorophyll *a* was extracted from the filters by a mixture of Acetone:Water 9:1 (v:v) and the extracts analyzed by absorption spectrometry following a modification of Lorenzen’s approach^49,50^ using a ThermoFisher Scientific Evolution 201 UV–Visible Spectrophotometer. The analysis of the dissolved nutrients was performed by UV/Vis spectrometry using specific colorimetric methods implemented in a Skalar SANplus Segmented Flow Auto-Analyzer specially engineered for the analysis of saline waters, considering separate lines for nitrate + nitrite (NO_3_^−^ + NO_2_^−^), nitrite (NO_2_^−^), ammonium (NH_4_^+^), phosphate (PO_4_^3−^) and silicate (Si(OH)_4_). NO_3_^−^ + NO_2_^−^ and NO_2_^−^ were determined according to Strickland and Parsons^51^, NH_4_^+^, Si(OH)_4_ and PO_4_^3−^ was determined according to Murphy and Riley^52^. All procedures were adapted to segmented flow analysis.

Measurements were performed by procedures adjusted and validated for the analysis of saline waters. Their quality was controlled through the analysis of blank samples, control standards close to the Limit of Quantification (LoQ), and in the middle of the calibration interval, duplicate samples and spiked samples, when applicable. Metrologically sound criteria were used to accept these controls^53,54^. The majority of measurements are accredited by the Portuguese Accreditation Body, IPAC, following the ISO/IEC 17025:2005 standard^55^ for the determination of these parameters in saline (estuarine and marine) water and those not under the scope of accreditation were submitted to the same rigorous quality control. For the LoQ and estimates of analytical uncertainty for the reported measurements (*U* and *U′*—absolute and relative expanded uncertainty for approximately 95% confidence level using a coverage factor of 2) please see the Supplementary Materials (Table S2).

### Data analysis

In order to assess the temporal and spatial variability in the estuary, the water quality parameters were compared considering the sampling stations, the month of the data collection, the tidal phase (High Water (HW), Ebb, Low Water (LW) and Flood) and the tidal amplitude (Neap Tides (NT) and Spring Tides (ST)). For such analysis, a PERMANOVA was performed using the PRIMER 6 + PERMANOVA software. In detail, data were normalized, *i.e*., each value of a parameter was subtracted by the mean and divided by standard deviation. Using the normalized data, a resemblance matrix was built with the calculation of the Euclidean Distances and an unrestricted model PERMANOVA with 9999 permutations was applied to this matrix. Due to the high influence of the spatial variability over the environmental data and in order to better assess the influence of the seasonal and tidal variations, the factors “month”, “tidal phase” and “tidal amplitude” were nested on the sampling stations. Moreover, due to the great internal variation of each tidal cycle (mainly between flood and ebb), the factor “tidal phase” was set as random while the remaining were set as fixed. Also, due to unbalanced design resultant of this type of sampling, a Type II sum of squares was applied. Pairwise tests were used as post-hoc tests and Principal Component Analysis (PCA) were used to explore the significant differences over the several parameters in study. PCAs were made by grouping the samples collected under NT and ST and identifying them according to the month and station in which they were obtained. Secondly, the samples were also grouped according to the station in which they were collected and classified according to the amplitude of the tide. Additionally, a cluster analysis was performed for the PCA, to aggregate similar data and facilitate a less subjective interpretation of the PCA. For PERMANOVA results and PCA statistics and eigenvectors, please see Supplementary Materials (Tables S4 to S11).

To further analyze the semidiurnal tidal variability, the water bodies (WB) defined under the Water Framework Directive (WFD) for the Sado Estuary^33^ were considered. WB1 encompasses stations A and B. Stations C and D were framed within WB3 and WB5, respectively.

## Results

**Figure 2:**
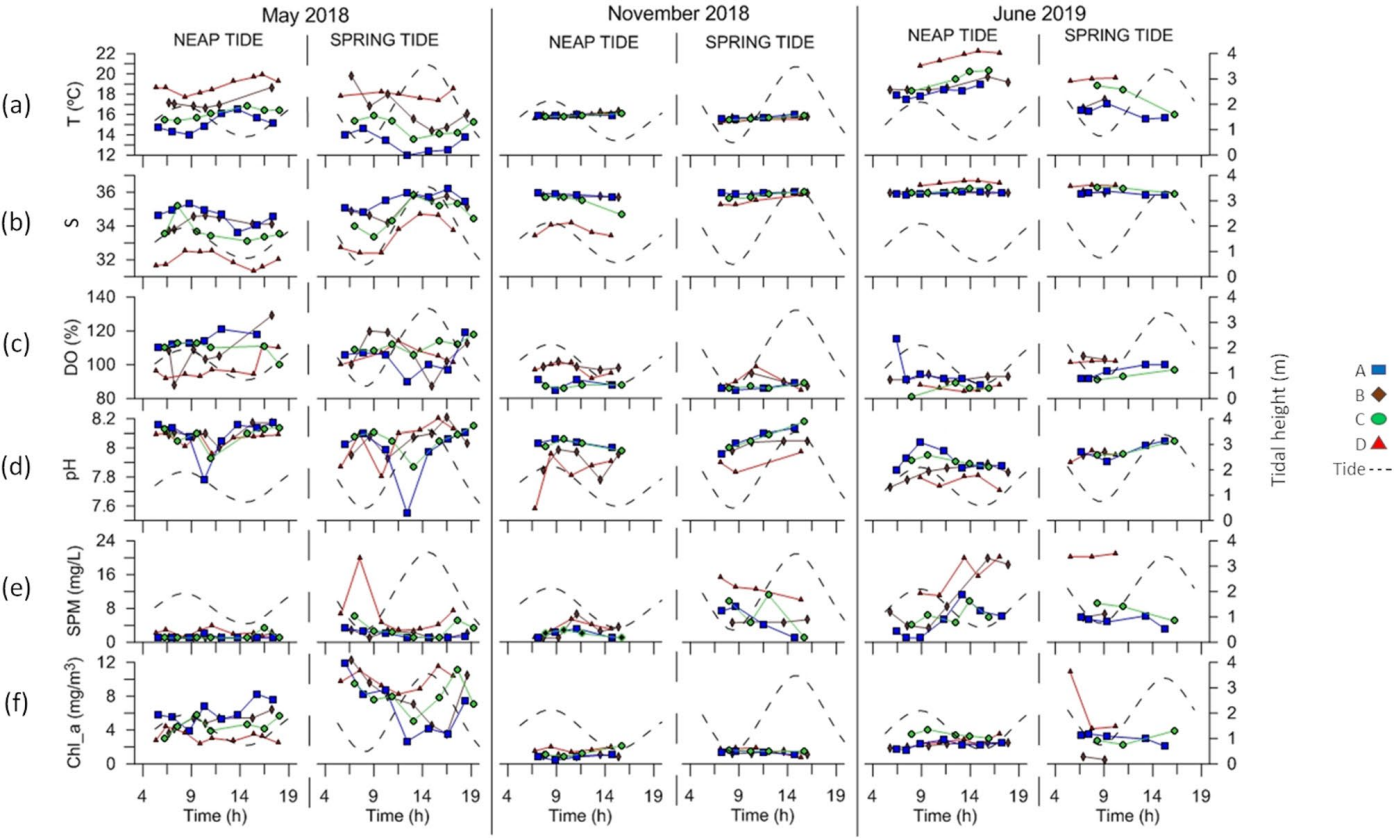
Tidal variability of the temperature (T, °C), salinity (S), dissolved oxygen (DO, %), pH, suspended particulate matter (SPM, mg/L) and chlorophyll *a* (Chl_*a*, mg/m^3^) observed during the sampling campaigns in neap tide and spring tide conditions. All data were collected at the surface of the water column. The tidal height is represented in grey dashed lines. Sampling stations are colored as follows: A—blue, B—brown, C—green and D—red. The figure was produced using PRIMER 6 (6.1.16) + PERMANOVA (1.0.6) software from PRIMER-e (https://www.primer-e.com/).

**Figure 3:**
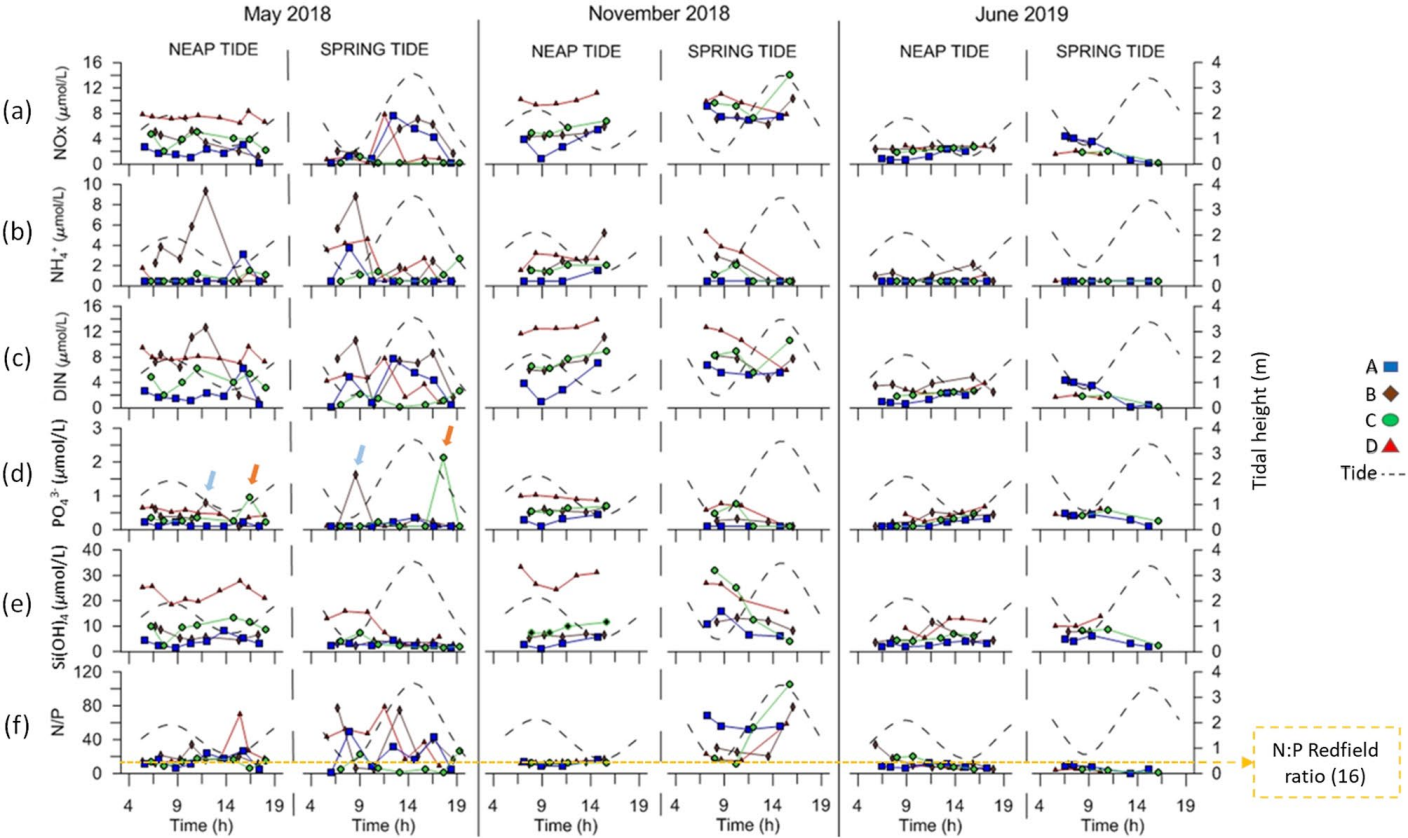
Tidal variability of the total oxidized nitrogen (NO_x_), ammonium (NH_4_^+^), dissolved inorganic nitrogen (DIN), phosphates (PO_4_^3−^), silicate (Si(OH)_4_) and nitrogen-phosphorus ratio (N/P) observed during the sampling campaigns in neap tide and spring tide conditions. All data were collected at the surface of the water column. The tidal height is represented in grey dashed lines. Yellow dashed line represents the N:P Redfield ratio (16). Blue and orange arrows correspond to unusual higher values of PO_4_^3−^ obtained at station B and C, respectively. Sampling stations are colored as follows: A—blue, B—brown, C—green and D—red. The figure was produced using PRIMER 6 (6.1.16) + PERMANOVA (1.0.6) software from PRIMER-e (https://www.primer-e.com/).

The variability of each parameter was analyzed over the semidiurnal tidal cycle in the different sampling stations, considering conditions of neap (NT) and spring tides (ST), at different times of the year (May/18, Nov/18 and Jun/19). The results of the sampling campaigns are shown in Figs. 2 and 3. The influence of the different variability factors considered, *i.e.*, spatial, temporal and tidal variability (semidiurnal and fortnightly) are described below.

### Spatial and seasonal variability

**Figure 4:**
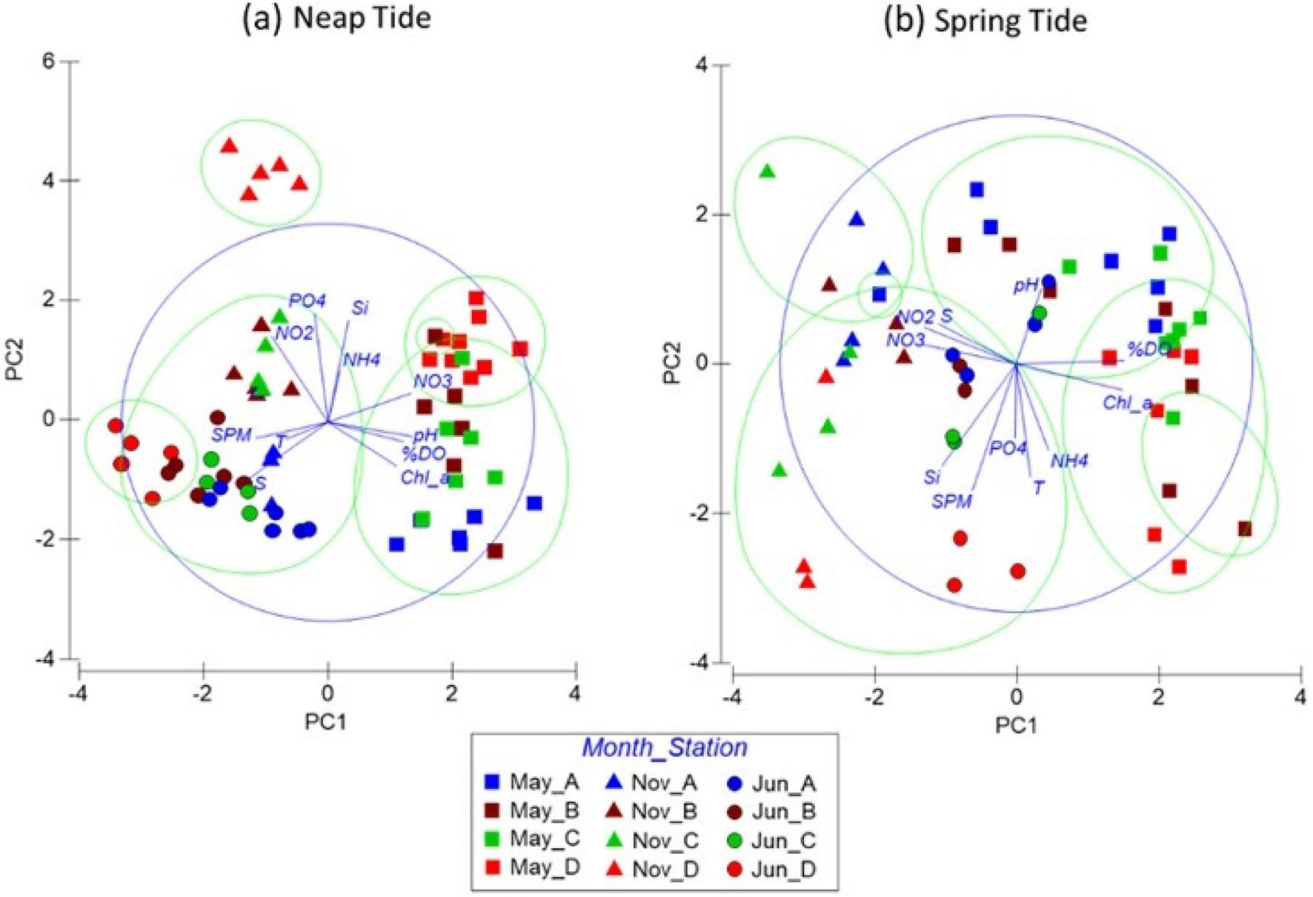
Principal component analysis (PCA) considering the data collected under neap tide and spring tide conditions. Samples classified according to the month of the data collection (May/18, Nov/18 and Jun/19) and the sampling station. *T* temperature, *S* salinity, *%DO* dissolved oxygen, *SPM* suspended particulate matter, *Chl_a* chlorophyll *a* and *Si* silicate Si(OH)_4_. The figure was produced using PRIMER 6 (6.1.16) + PERMANOVA (1.0.6) software from PRIMER-e (https://www.primer-e.com/).

Considering all the parameters analyzed in this study, significant differences were observed among sampling stations through a PERMANOVA analysis (p-value = 0.0001; Figs. 2 and 3; Table S4) The pairwise post-hoc test, used to relate the sampling stations, revealed the existence of a clear spatial variability along the estuary, with all stations showing significant differences among themselves (p-value < 0.05; Table S5), except between stations B (near Setúbal) and C (south channel of the estuary), which showed similarities throughout sampling (p-value = 0.0992; Table S5). It was also possible to conclude that there was a significant difference between the sampled months in all stations, suggesting the existence of seasonal variability (p-value = 0.0001; Table S4). PCAs showed that seasonality was the most influencing factor under NT conditions, while under ST, the spatial differentiation was more evident (Fig. 4 and Tables S6 and S7; together PC1 and PC2 covered 58.2% and 53.6% of the variation existing in the water samples under NT and ST conditions, respectively, with PC1 representing 33.8% and 35.0% of this variation). Overall, the greatest spatial variability was observed in the campaigns carried out in May/18 (Figs. 2 and 3).

Exception was for the SPM, which presented the highest spatial variability in Jun/19. From the data set, it was possible to observe some clear spatial patterns for most of the parameters (Table 1). However, unusual variations observed in some campaigns and stations (Figs. 2 and 3) were worth mentioning and are described below.

**Table 1:** Range of values observed for each parameter and the respective average, considering the data obtained during the six campaigns for each sampling station.

	Station A			Station B			Station C			Station D		
	Min	Max	Mean	Min	Max	Mean	Min	Max	Mean	Min	Max	Mean
T (°C)	12.0	19.0	*15.6*	14.4	19.8	17.0	13.6	20.4	16.3	15.2	22.2	**18.4**
S	34.0	36.2	**35.6**	33.7	36.0	35.4	33.3	36.3	35.1	31.3	36.7	*34.1*
DO (%)	85.0	121.0	99.2	87.0	129.0	**100.6**	81.0	118.0	98.4	84.0	114.0	*96.8*
pH	7.6	8.2	**8.0**	7.7	8.2	**8.0**	7.9	8.2	**8.0**	7.6	8.2	*7.9*
SPM (mg/L)	1.0	11.2	*3.1*	1.0	19.8	4.3	1.0	11.2	4.0	1.1	20.9	**7.8**
Chl_*a* (mg/m^3^)	0.4	11.9	*3.7*	0.5	12.3	*3.7*	0.9	11.2	4.2	0.7	11.6	**4.3**
NO_x_ (μmol/L)	0.4	8.5	*2.7*	0.4	7.9	3.9	0.4	10.6	3.3	0.4	11.2	**5.3**
NH_4_^+^ (μmol/L)	0.5	3.7	*0.8*	0.5	9.3	**2.5**	0.5	2.7	1.0	0.5	5.3	1.6
PO_4_^3−^ (μmol/L)	0.5	0.1	*0.2*	0.1	1.6	0.4	0.1	2.1	0.4	0.1	1.0	**0.5**
Si(OH)_4_ (μmol/L)	1.1	16.0	*4.0*	2.2	13.2	6.1	1.4	31.8	8.1	2.0	33.2	**17.8**

In bold and italics are the highest and lowest average values for each parameter, respectively.

The region of the mouth of the estuary (station A) usually showed the lowest temperatures (Table 1). Even though high salinity values were observed along the estuary (31.3–36.7, Table 1), it was also in this region that the highest average salinities were observed. A clear pH spatial pattern was not perceived. Particularly in May/18, a sharp decrease was observed in the pH at the mouth of the estuary (Fig. 2d). Throughout the sampling period, the mouth of the estuary also usually presented the lowest chlorophyll *a* values and the lowest nutrient concentrations, with some exceptions (*e.g.*, NT in May/18 for chlorophyll *a*). Although this region tended to present the lowest DIN, in Jun/19 (ST) presented slightly higher values than the inner region of the estuary (probably due to the high NO_x_) (Fig. 3c). Overall, station A presented low values of PO_4_^3−^, NH_4_^+^ and Si(OH)_4_ throughout sampling, as also revealed by the PCA (Fig. 4).

Sampling station D, the most inner station of the estuary, was the most contrasting. As illustrated by the PCA, under NT, station D presented high nutrient concentrations in May/18 and Nov/18, while in Jun/19, it showed high SPM and temperatures (Fig. 4). It was in the inner area of the estuary that the lowest salinity values were usually detected. On the other hand, this same area presented the highest salinity values in Jun/19, mainly under NT (Figs. 2b, 4). In the inner area of the estuary, the highest chlorophyll *a* and NO_x_ average values were observed (Table 1). However, exceptions occurred, for example in Jun/19, station D presented the lowest values of NO_x_ (≈ 2.0 μmol/L), which matched with the particularly higher chlorophyll *a* values also observed (Fig. 2f).

The inner and outermost regions of the estuary tended to show opposite behaviors in the variability of the parameters and that was mainly evidenced in the PCA obtained under ST (Fig. 4). However, this was not always observed. Station B, located near the city of Setúbal, showed a distinct behavior from the remaining stations regarding NH_4_^+^ variability, presenting the highest NH_4_^+^ values (Table 1). Still, in Nov/18 (ST), the highest NH_4_^+^ values were observed in station D (Fig. 2c). The highest DO average value (Table 1) was also observed near the city of Setúbal. Overall, the DO was higher than 80% in all samples, often exceeding 100%, as expected because the data refer to surface values quantified during daylight. Although with small differences, the most inner stations of the estuary presented higher PO_4_^3−^ values (this pattern was not observed in May/18, in the ST campaign—Fig. 3d). In May/18, a PO_4_^3−^ peak was observed at stations B and C (blue and orange arrows in Fig. 3d, respectively). These irregular values were obtained under different tidal conditions, showing that they were not derived by the tidal variability. In both campaigns, the peaks were observed in the morning in station B, near Setúbal, and at the end of the day in station C, near Troia.

### Fortnightly tidal variability: neap tides vs spring tides

It was also possible to conclude that the variation between NT and ST significantly affected the quality of the water in almost the whole estuary (p-value = 0.0001, PERMANOVA test) (Table S4). According to the pairwise post-hoc test, this influence was more significant in station D, the most innermost station analyzed (p-value = 0.0001; Table S8). However, the parameters did not vary significantly with the fortnightly tidal cycle in station C, located in the south channel of the estuary (p-value = 0.1642; Table S8). From the PCA (Fig. 4), a greater dispersion of the data were observed along the estuary under ST conditions, revealing a higher influence of several parameters. Although significant differences between NT and ST were detected also at stations A and B, it was difficult to detect clear patterns over time. However, some of the parameters showed greater evidence of the influence of the fortnightly tidal cycle in their variability. Station A was associated with lower temperature values under ST, during the whole sampling period, and high values under NT, particularly in Nov/18 and Jun/19 (PCA, Fig. 4). The campaigns conducted in Nov/18 showed the highest differences between the SPM samplings conducted under NT and ST conditions (Fig. 2d), with higher values observed under ST. Particularly during the campaigns conducted in May/18, higher chlorophyll *a* concentrations were obtained under ST along the estuary (Fig. 2f).

**Figure 5:**
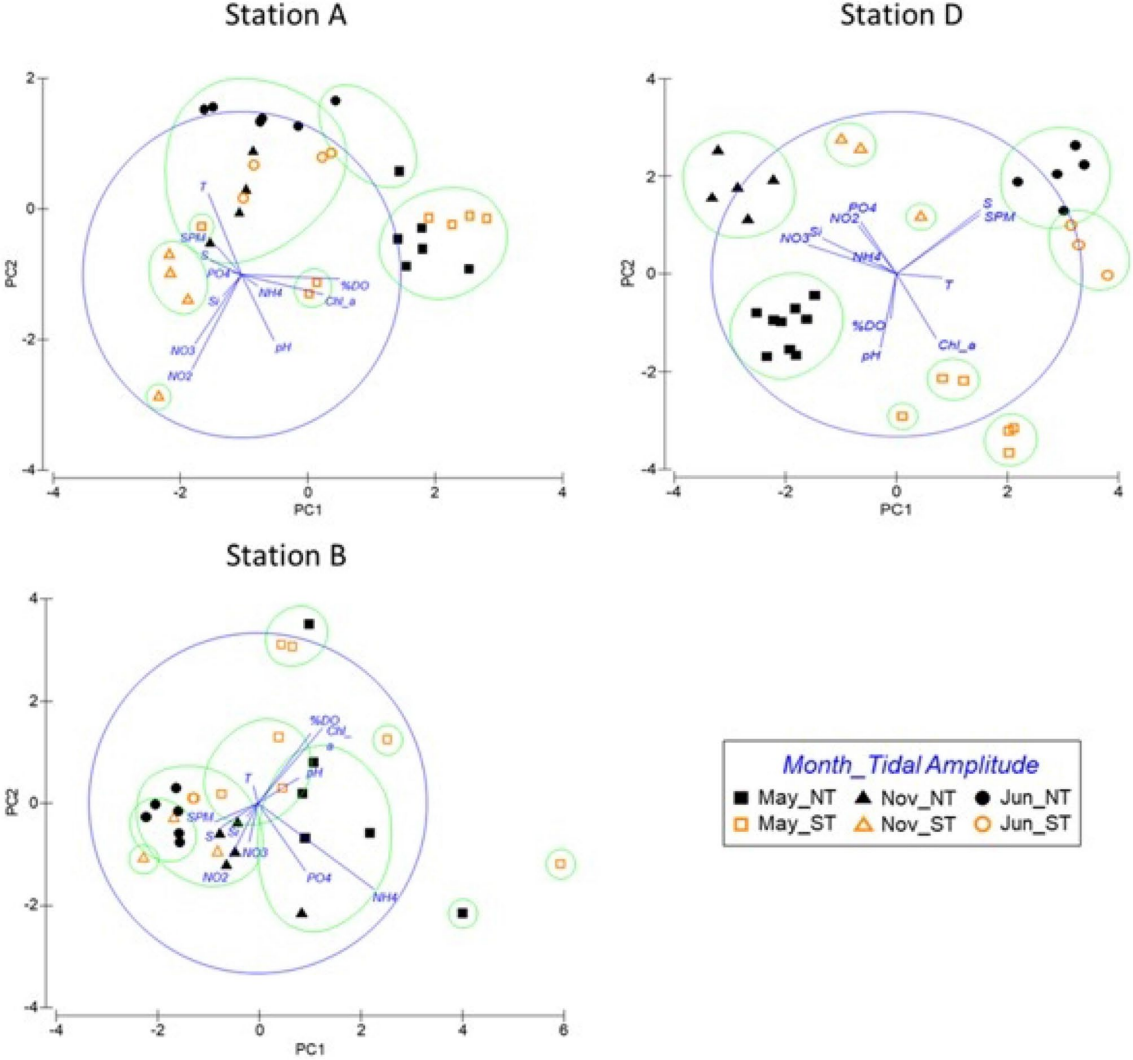
Principal component analysis (PCA) considering the data collected in the sampling stations A, B and D. Samples classified according to the month of the data collection (May/18, Nov/18 and Jun/19) and the respective tidal amplitude (NT and ST). *T* temperature, *S* salinity, *%DO* dissolved oxygen, *SPM* suspended particulate matter, *Chl_a* chlorophyll *a* and *Si* silicate Si(OH)_4_. The figure was produced using PRIMER 6 (6.1.16) + PERMANOVA (1.0.6) software from PRIMER-e (https://www.primer-e.com/).

According to the PERMANOVA analysis, a significant influence of the fortnightly tidal cycle on the variability of the parameters in station A, located at the mouth of the estuary, was also observed. This is probably the result of the high values of temperature and SPM concentrations, as well as lower NO_x_ concentrations obtained under NT in Nov/18 and Jun/19 (Fig. 5, Table S9—PC1 and PC2 represented 59.0% of the observed variation, with PC1 representing 41.2%). At station B, the main differences observed between NT and ST, seemed to occur in May/18, where NT presented, in general, higher NH_4_^+^ and PO_4_^3−^ than ST (PCA, Fig. 5, Table S10—PC1 and PC2 together accounted for 60.6% of the variation observed in the samples, being the PC1 responsible for 39.7%). Only station D presented a clear segregation between these two tidal conditions, with the cluster analysis of the PCA clearly isolating the data collected under NT (Fig. 5, Table S11—PC1 and PC2 encompassed for 72.4% of the observed variation, being PC1 = 42.9%).

### Semidiurnal tidal variability: low water vs high water

Through the PERMANOVA analysis it was also possible to see that the semidiurnal tidal cycle significantly influenced the variability of the parameters (p-value = 0.0124, Table S4). However, from the set of factors considered in the analysis, it was the factor that least influenced the variability of the water quality in the estuary. The incidence of the observed significance was assessed for each one of the sampling stations and only stations B and C showed a significant influence of the semidiurnal tidal cycle on the variability of the parameters.

Overall, the greatest amplitude of values along the tidal cycle was obtained in the campaigns conducted in May/18. In Nov/18 and Jun/19, the variability of parameters over the tidal cycle was much lower in this region of the estuary. Even so, general patterns in the variability of some parameters according to the semidiurnal tidal cycle were observed (Figs. 2 and 3). The significant differences between tidal phases were not perceptible through the PCA analysis (Figs. 4 and 5).

Along the estuary and throughout the campaigns, higher temperatures were usually observed during ebb, with the highest values being observed near LW (Fig. 2a). Salinity and pH seemed to vary along with the tidal cycle: higher values were observed during flood and lower values during ebb (as opposed to the temperature variability) (Fig. 2b,d). The SPM values appeared to decrease during flood (maximum values around LW) (Fig. 2e). This pattern was also occasionally observed for the chlorophyll *a*, but it was not clear for all the campaigns nor for all the sampling stations, as for station C in Jun/19 (Fig. 2f). It was difficult to associate the variation of the NO_x_ with the semidiurnal tidal variability. However, occasionally, the values increased with flood, as occurred in the campaign conducted in May/18 under ST conditions, with evidence at stations A and B, which, again, also presented lower chlorophyll *a* values (Fig. 3a). PO_4_^3−^ variability could be associated with the semidiurnal tidal variation: the lowest values were observed close to high water (except in May/18). Si(OH)_4_ was the nutrient presenting the highest concentrations (< 0.3–35.0 μmol/L, Fig. 3e and Table 1). Its variation was linked to tidal variability, with a decrease of the values observed during flood as well (Fig. 3e). In May/18, particularly under ST, N/P mostly varied between 20 and 80 along the semidiurnal tidal cycle, indicating a possible phytoplankton limitation by phosphorus (Fig. 3f). On the other hand, in Nov/18 (ST) and Jun/19, there was a small variation of the N/P values along the tidal cycle and throughout the estuary, with most of the samples showing values ≈ 10, indicating a possible limitation of phytoplankton biomass by nitrogen concentration.

### Relationships between parameters

**Figure 6:**
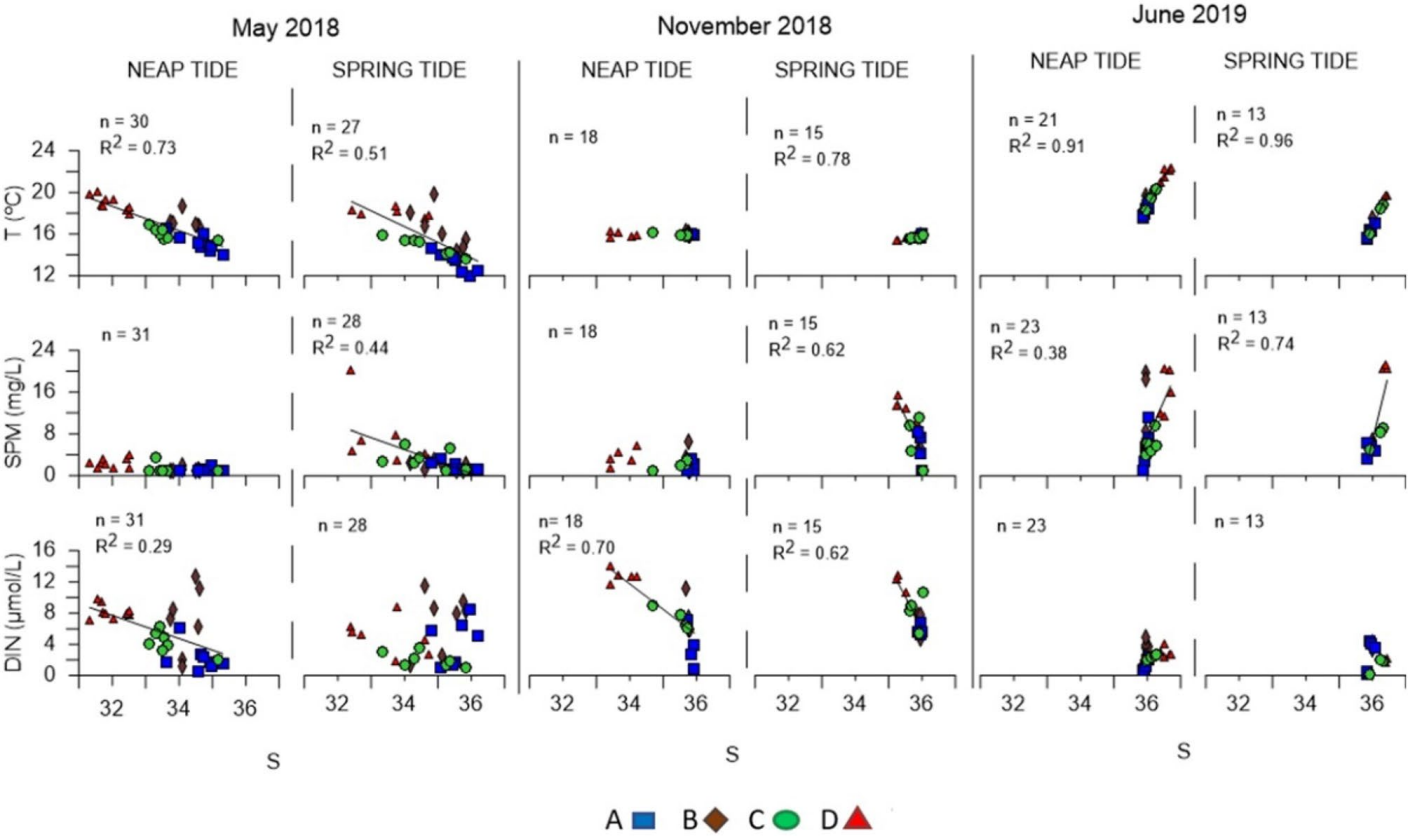
Relationships between temperature (T), suspended particulate matter (SPM) and dissolved inorganic nitrogen (DIN) with salinity, observed during the sampling campaigns in neap tide and spring tide conditions. Data collected at the surface. Only R^2^ > 0.2 and the respective lines are presented. The figure was produced using PRIMER 6 (6.1.16) + PERMANOVA (1.0.6) software from PRIMER-e (https://www.primer-e.com/).

To a better understanding of how the different parameters were associated and how their variability was conditioned by other variables, relationships between some parameters were assessed. Firstly, the temperature, SPM and the DIN values were combined with the respective salinity data (Fig. 6). In Fig. 6, only the coefficients of determination (R^2^) showing linear relations R^2^ > 0.2 between the parameters are presented.

It was observed that the temperature and the salinity data were related in different ways throughout the campaigns (Fig. 6). In May/18, higher salinities were associated with lower temperatures, showing moderate to high relationships (R^2^ = 0.73 and R^2^ = 0.51 for NT and ST, respectively). The lowest temperatures were observed near the coast and the highest ones in the most inner station of the estuary. In Jun/19, the inverse relationship was observed (R^2^ = 0.91 under NT and R^2^ = 0.96 under ST), indicating that the interior of the estuary was warmer and lightly saltier. During the ST campaigns conducted in Nov/18 the relative reduced range of salinity values was framed in practically unchanging temperatures (R^2^ = 0.78).

Lower SPM appeared to be related with higher salinities in the campaigns conducted under ST in May/18 and Nov/18 (R^2^ = 0.44 and R^2^ = 0.62, respectively), mainly near the mouth of the estuary, revealing a riverine origin of this last parameter. In the campaigns conducted in Jun/19, higher salinities were observed in the whole estuary (R^2^ = 0.38 and R^2^ = 0.74 for NT and ST, respectively). DIN only showed a high relation with salinity in the campaigns carried out in Nov/18, with higher DIN values associated with riverine waters, characterized by lower salinities (R^2^ = 0.70 under NT and R^2^ = 0.62 under ST). This pattern was like the one observed in May/18 (mainly under NT). Figure 6 also shows the spatial variability of the parameters. The mouth of the estuary (station A) usually showed the lowest temperatures, SPM and DIN, in opposition with the most inner region of the estuary (station D). In May/18 and Nov/18, higher salinities were registered the mouth of the estuary but in Jun/19 it was the inner region of the estuary that yielded the highest values.

**Figure 7:**
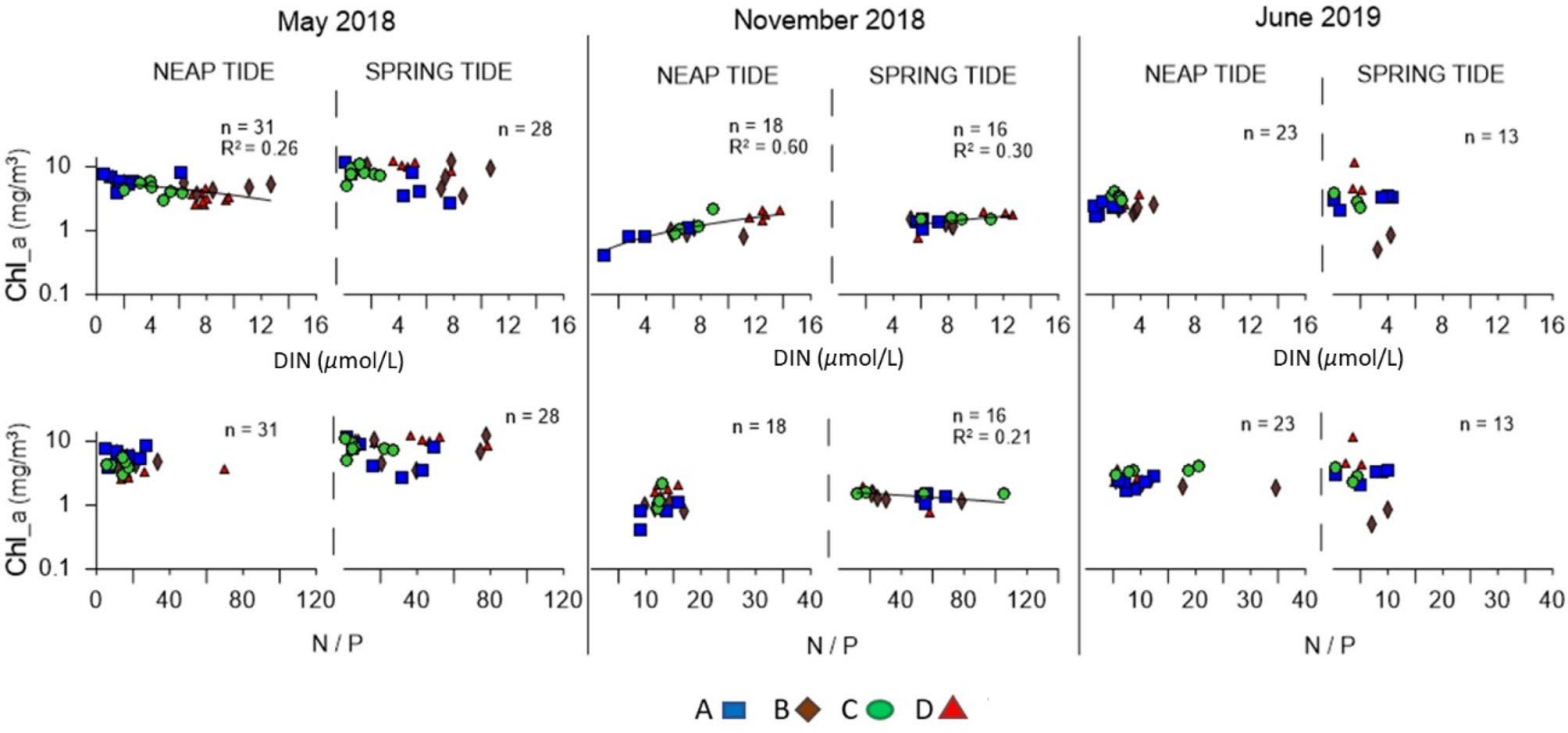
Relationships between the chlorophyll *a* and the dissolved inorganic nitrogen (DIN) and the N/P ratio, observed during the sampling campaigns in neap tide and spring tide conditions. Data collected at the surface. Chlorophyll *a* in log10 scale. Only R^2^ > 0.2 and the respective lines are presented. The figure was produced using PRIMER 6 (6.1.16) + PERMANOVA (1.0.6) from PRIMER-e (https://www.primer-e.com/).

Chlorophyll *a* was also matched with DIN and the N/P ratio (Fig. 7). Once more, only the R^2^ > 0.2 are presented. In May/18, higher chlorophyll *a* concentrations were observed, and they were related to lower DIN values (R^2^ = 0.26). In Nov/18, the highest chlorophyll *a* concentrations were associated to the highest DIN concentrations and were observed in the most inner stations of the estuary. In Jun/19, no relevant relationship was observed. In Nov/18 (NT) and in Jun/19, mainly in ST, most of the N/P values were below 16 (Fig. 3f), suggesting a limitation of N, which is corroborated by the low concentrations of DIN (for an estuary) also observed.

## Discussion

### Spatial and seasonal variability

The sampling stations showed significant differences among themselves, revealing a noticeable spatial variability of the water quality parameters in the Sado Estuary. Only stations B (located near Setúbal) and C (in the south channel of the estuary, near Troia), showed similarities during sampling. This spatial differentiation has been described in the available literature^11,12,28,33,37^ and clearly influences the variation of the parameters during the semidiurnal tidal cycle, as mentioned earlier.

In general terms, the spatial patterns obtained through the present study were in accordance with the results of previous studies. The estuary showed salinity values higher than 25 along the estuarine area, behaving like a coastal-lagoon^33^. The most inner station (station D) frequently presented higher values for the whole set of parameters than the ones observed at the mouth of the estuary, with exception of the dissolved oxygen, pH and the salinity, which were usually lower in that area^10^. Sent et al.^11^ also observed the highest SPM concentrations in the inner channels of the estuary. Regarding salinity, the highest values were observed in the inner area of the estuary in Jun/19 (associated to high SPM). This pattern may be justified with the high evaporation that occurs in that region of the estuary during Summer^12^. This inner evaporation-driven salinity under dry weather is also observed in other coastal-lagoon low-inflow estuaries, such as Urias Estuary (Gulf of California)^56^, but also in tropical estuaries, as the mainland Australian ones^57^. During some of the campaigns, mainly in May/18, the highest values of ammonia were observed at station B, near the city of Setúbal. This spatial differentiation could be related with anthropogenic activities derived from the main urban center of the estuary; this result corroborates the conclusions of Gonçalves et al.^12^ and it also observed in several mainland estuaries of the United Kingdom^58^. During the campaigns Nov/18 and Jun/19 (ST), different values of DO were observed along the two routes of water exchange (north and south channels): at stations A and C, the values were lower than the ones obtained at stations B and D, located in the south channel. Gonçalves et al.^12^ also detected this differentiation between the channels but presented higher values for the south channel.

The values of chlorophyll *a* observed in the inner area of the estuary presented slightly higher values than the mouth. However, in the first campaign conducted in May/18 (NT), the highest values were recorded at the mouth of the estuary, and this pattern was reversed in the campaign carried out a week later (ST). Under ST, the higher concentrations observed were probably originated in the inner part of the estuary, where the phytoplankton biomass possibly increased due to the resuspension of the benthic communities^59^, which can happen more easily with the stronger currents observed under ST^60^. The high chlorophyll *a* concentrations detected at the mouth of the estuary in May/18 (NT) stood out from the remaining data, indicating an external influence. This could be a consequence of coastal upwelling but needs further research. In general terms, coastal upwelling occurs along the west coast of Portugal during spring and summer but seems to not affect the inner Sado Estuary^37,61^, that is located in a “shadow” area. However, in case conditions are favorable (*e.g.,* wind and currents intensity and direction), it may be a possibility, mainly in the mouth of the estuary. In fact, a preliminary analysis using remote sensing sea surface temperature data, showed the occurrence of coastal upwelling in the region during this period. Also, microscopy analysis of the phytoplankton community revealed the presence of coastal species in the outermost region of the estuary (data not shown), which seems to corroborate the hypothesis. Coutinho^37^ verified that, in the Sado Estuary, the highest values of chlorophyll *a* usually occur during spring and summer (as, for example, in Tampa Bay (Florida)^62^ or Kuala Sibuti Estuary (Malaysia)^63^). The low chlorophyll *a* values, observed in the campaigns carried out in June/19, could reveal that the seasonal maximum of chlorophyll *a* had already taken place, with the concentrations gradually decreasing afterwards.

### Fortnightly tidal variability: neap tides vs spring tides

There are records of the influence of the fortnightly tidal cycle in the water quality of estuaries worldwide (e.g., Fortune and Mauraud^15^; Fatema et al.^64^; Tufoni^16^) and, accordingly, the present study showed that the variation of the environmental parameters analyzed was influenced by NT and ST conditions, particularly in stations A, B and D.

This influence was more significant in station D, the innermost station of the estuary. From the complete set of four stations, station D was the least influenced by the sea and more by the Sado River, which was showed through the spatial variability of the salinity. This allows greater variations under spring tide conditions, due to the stronger sea-riverine variability influencing the water quality parameters^65^, in opposition with the higher stability under neap tide conditions. In station C, located in the south channel, no significant variations in the parameters were observed at all the temporal time scales studied. This could be related with the circulation regime of the estuary. Station C was located in the main route for water exchange in the estuary, where high intensity currents were observed, both during flood and ebb^24^. The occurrence of those strong currents could mitigate the influence of the variation between NT and ST. However, further studies should be conducted to confirm this. The assessment of the spatial variability of the tidal influence in the environmental parameters of estuarine systems is not commonly mentioned in the existent bibliography.

Studies conducted in the Tagus Estuary (Portugal), the closest to the Sado Estuary, concluded that during spring tide, the SPM increase, while the chlorophyll *a* concentrations decreased according to a model published by Vaz et al.^66^. During NT, the opposite occurred: SPM decreases while the chlorophyll *a* concentration increases. This increase in SPM, under ST conditions, was also observed in another Portuguese estuary—the Guadiana Estuary^67^. In the present study, SPM was higher under ST in May/18 and Nov/18, observation supported by the mentioned studies, but not in June.

Fatema et al.^64^ showed that some parameters such as temperature, salinity, nitrate, ammonia, DN and DP yielded higher values during ST, while DO, pH and nitrite were higher under NT, in the Merbok Estuary (Malaysia). Likewise, Station A was associated with lower temperature values under ST in the present analysis. Other works presented different patterns, with higher temperatures observed under NT (e.g., Gonçalves et al.^21^ but they were the result of a clear variability detected along the entire estuary. Regarding other parameters, these kind of variability was not easily detected in the present analysis, and that could be justified by the conditions in which the sampling campaigns took place. Lower variability of the parameters over time and along the estuary occurred during Nov/18 and Jun/19. These campaigns were conducted under adverse weather conditions, with heavy rain and rough seas. This could have led to a greater mixing of the water column, masking the differences due to tidal amplitude, resulting in the observed low temporal variability. As mentioned by DiLorenzo et al.^14^, the weather conditions are causes for variability of the parameters and they influence the water quality analysis under different tidal conditions.

In the future, it would be also important to consider the entire water column and not use only surface observations, to understand how the water column mixing is affected by the fortnightly tidal cycle. For example, the Tagus Estuary (Portugal) can generally be classified as partially-mixed, but when neap tide conditions combine with high river discharge, it becomes stratified^68^. Sampling in the upstream area of the estuary should be considered too.

### Semidiurnal tidal variability: low water vs high water

**Figure 8:**
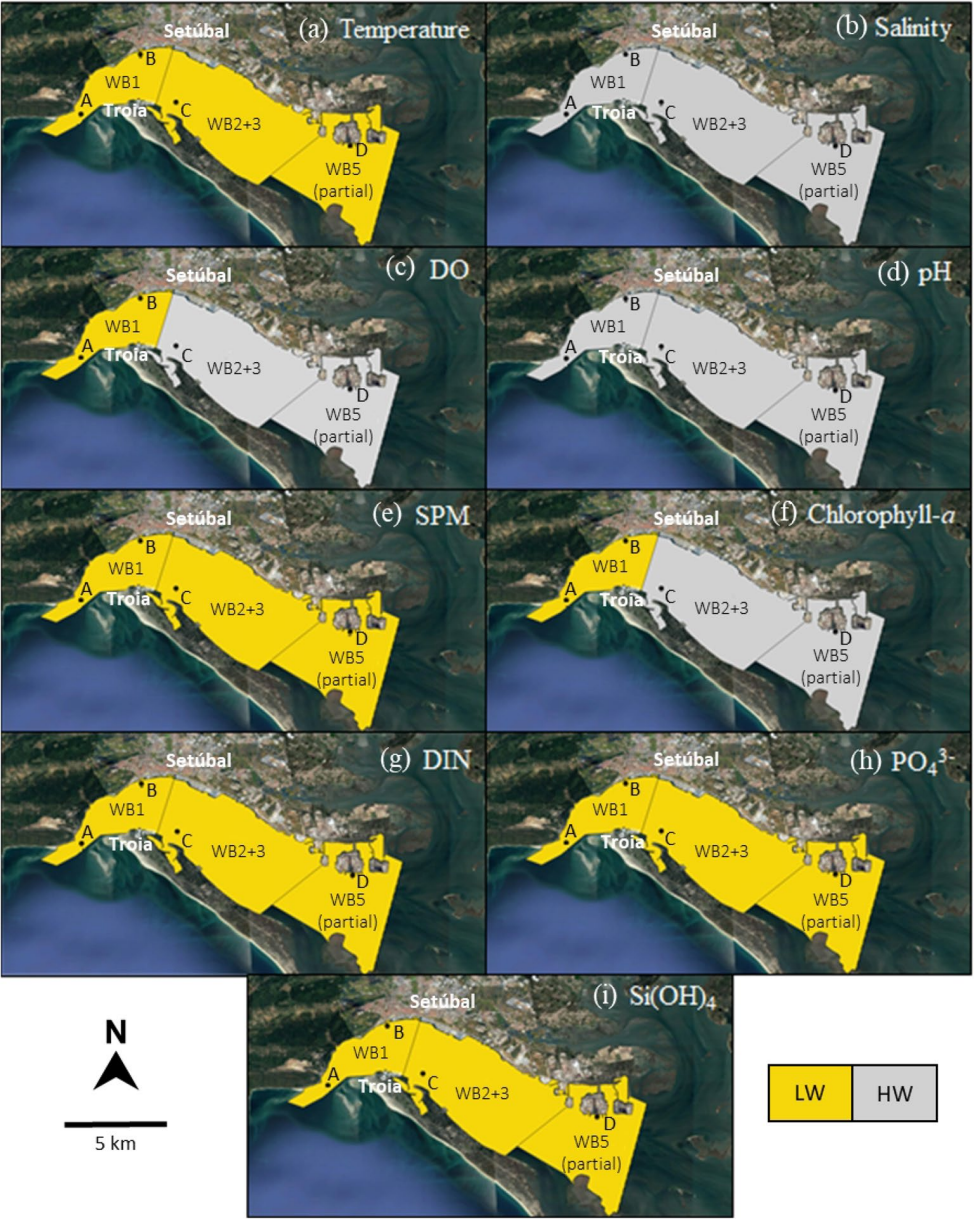
Tidal phase in which the highest average value of the various parameters was observed in the different areas of the estuary considering the data collected in stations A, B, C and D. Low Water (LW) is represented in yellow and High Water (HW) in grey. Water Body definition, currently used in the context of the WFD, followed Ferreira et al.^33^. Note that WB2 and WB3 are represented together (WB2 + 3) and WB5 is only partly represented. The maps were produced using QGIS 3.8.3 Zanzibar, from QGIS Association (https://www.qgis.org/).

The semidiurnal tidal cycle was an important driver in the behavior of some parameters, showing a significant influence on the water quality at stations B and C, located near Setúbal and in the south channel (near Troia), respectively. Figure 8 shows the tidal phase corresponding to the occurrence of the highest average value for each one of the observed parameters, considering the different areas of the Sado Estuary: LW in yellow and HW in grey. The regions considered were based on the water bodies (WB) defined under the Water Framework Directive (WFD) applied to the Sado Estuary^33^. Due to the lack of data in the WB2, that region was assumed to have the same behavior as WB3. WB5 is only partially represented due to the lack of data in the innermost region of the estuary (spatial limitation). Information about the average values used in this study, are present in Table S12 of the Supplementary Materials.

The parameters with higher values at LW, clearly show a greater influence of the river flow. One of those parameters was the temperature, as it was also described by Gonçalves et al.^12^ and Cereja et al.^69^ in the Sado Estuary. This observation is one more indicator that the Sado River presents higher temperatures than the coastal ocean^70^, in opposition to what was mentioned in previous studies conducted in the Sado Estuary at the same time of the year^71^. These lower temperature values were concomitant with the lowest SPM concentrations_._ In the existing literature, there are records of SPM levels increasing during flood in the Guadiana Estuary^67^, the Tagus Estuary^72,73^ or the Botlek Harbour, Rotterdam Waterway^74^. In these cases, the ocean induces resuspension of bottom sediments and increases the SPM concentration in the water column^72^. Less frequently, the concentrations of SPM increase during ebb, as in the Arade Estuary^18^ and in the present study. The river flow and the surface runoff could also enhance the increase of suspended particulate matter in the water. This set of factors can lead to an increase of SPM during the ebb in the Sado Estuary, where higher values are usually observed in the inner area^75^.

In estuaries, the SPM variability is strongly related to the variation of the nutrient concentrations, as SPM can desorb and absorb nutrients and influence the primary production^76^. In the present study, higher values of Si(OH)_4_ and DIN were also observed at LW, which evidenced this relation. This tendency can be an indicator of the riverine origin of the nutrients in the estuary. Data collected within the project also show that there is a clear gradient in nutrient concentrations that follows the salinity gradient, with higher concentrations usually associated with lower salinities, at the most inner stations (data not shown). In addition, benthic-pelagic coupling, more evident during to lower water level (or ebb), may strongly contribute to this increase in nutrients, especially at the most inner stations, where the water column is shallower^77^. Therefore, the estuary can be considered as a source for nutrients. Gonçalves et al.^12^ concluded that NO_x_ increased along the tidal cycle in the bay of the Sado Estuary, showing higher values at HW. In the present study, there was also some evidence of oceanic waters introducing higher concentrations of NO_2_^−^ + NO_3_^−^.

High DO values were recorded at LW mainly in May/18, when higher chlorophyll *a* concentrations were also observed. Figure 8 shows a chlorophyll *a* pattern similar to the one of DO along the estuary, suggesting that chlorophyll *a* in the outermost region of the estuary has a more inner origin. Possibly, the relationship between DO and phytoplankton biomass could be related to primary production, as observed by Iriarte et al.^78^ in the Bilbao Estuary. Although the salinity presented a low variability, higher salinity values were observed at HW, in all the sampling stations, throughout the campaigns, thus justifying the pH variation in the estuary. The inflow of oceanic waters into the estuary, during flood, increases the salinity and the pH content^10^. Overall, temperature, SPM, PO_4_^3−^, DIN, Si(OH)_4_ and chlorophyll *a*, in the outermost area of the estuary, seemed to be related and show an enhancement when the river water is the main driver of the system, *i.e*., during the ebb.

### Implications of the tidal variability in the water quality assessment

Under the Water Framework Directive (WFD), it is mandatory to assess the ecological status of the water bodies located in Portuguese territory, including the Sado Estuary^27^. For this, assessing the worst case-scenario seems like a sensible approach. This scenario would include the conditions under which the highest concentrations of nutrients and chlorophyll *a* are detected or the lowest levels of oxygen are measured, as these are some of the possible eutrophication symptoms that could lead eventually to poor water quality in the estuary^79^. The tidal cycle has an influence on the variation of those parameters and should be considered when sampling strategies are defined. From the present analysis, a tendency (albeit spatially and temporally inconstant) to higher values of chlorophyll *a* and nutrients was observed close to the LW. In fact, few guidelines are available to determine the potential benefit of collecting water quality data at a fixed tidal phase (low tide) and whether resources should also be directed towards mitigating other causes of variability such as precipitation^14^. However, if the purpose is to observe the worst-case scenario in water quality of the Sado Estuary, sampling at LW should be the most appropriate approach (even if some areas of the estuary would no longer be accessible), as also mentioned by Mallin et al.^25^. Moreover, in this case, the entire estuary should be monitored, as its downstream region is one of the most affected by anthropogenic presence, hence it should be also carefully analyzed. The results obtained can be easily transposed to other estuarine systems with similar hydrodynamics and morphological conditions (Robins et al.^80^; Scanes et al.^81^).

Regardless of the time of day or the phase of the tidal cycle, the surface water of the Sado Estuary can be considered well oxygenated^12^, with values higher than 80% in all samples, often exceeding 100%. Regarding chlorophyll *a*, within the scope of the WFD, boundaries were defined to classify the estuary's water bodies according to their ecological status. For salinities above 25, the reference value of chlorophyll *a* for the Sado Estuary is 6.67 mg/m^3^^82^. Overall, the values herein observed were lower than the reference, revealing a high ecological status of the system. In May/18 (ST) under low water, these values exceeded 10 mg/m^3^, the value from which the ecological status of the system could be considering worrying (moderate ecological status). Thus, the variation of the semidiurnal tidal cycle can influence the water quality of the estuary. Based on the relations between chlorophyll *a* and the DIN and chlorophyll *a* and the N/P (Fig. 7), there were no signs of eutrophication in the Sado Estuary^3^.

Following the reference values for nutrients defined in the Sado Estuary by Caetano et al.^28^ under the WFD (considering the recommended 1 to 25 salinity class), the water quality status was evaluated considering the observed maximum concentrations of NO_x_, NH_4_^+^ and PO_4_^3−^: 14.0 μmol/L, 9.5 μmol/L and 2.5 μmol/L, respectively. The ratio [nutrients]/reference values was lower than 1, indicating that the nutrients concentrations were lower than the imposed limits^28^. Regardless of the tidal phase, the estuary also showed high quality waters regarding its nutrient’s concentrations. However, Caetano et al.^28^ detected areas in the Sado Estuary, with medium quality, mainly due to high concentrations of PO_4_^3−^. As such, abnormally high values should always be carefully analyzed, as the unusually high NH_4_^+^ concentrations obtained near Setúbal (station B). In urbanized estuaries, one of the sources of NH_4_^+^ is the sewage effluent^58,83^, and these particularly high NH_4_^+^ concentrations were detected during ebb. Sewage effluent from part of the city of Setúbal is discharged directly into the estuary near station B. The works to divert the sewers to the Setúbal Wastewater Treatment Plant were not completed, by the time the sampling campaigns were finished^84^. High NH_4_^+^ concentrations could promote the primary production and can deteriorate the water quality^58^. However, during flood, these high concentrations were no longer observed, having been mitigated by the renewal of water of the estuary. This is an indicator that the tidal variability plays a fundamental role in regulating the water quality in the Sado Estuary and in mitigating the harmful effects of human activities. Therefore, land discharges should be made so that their effect on the estuary could be mitigated by the variation of the tidal cycle. It is also necessary to ensure that the discharges are legally regulated, to detect a cause-effect relationship between anthropogenic pressures and the biological component of the estuary, which could be translate in a quick response from phytoplankton to this type of pressure.

Overall, the results obtained showed a slight decrease in the water quality status in the Sado Estuary at low water compared to high water, which can be misinterpreted if the tidal variation is not taken into account. In this case, even considering the worst case-scenario, the final quality status would remain similar (with one exception). However, this study only integrates specific snapshots of the estuarine conditions. Thus, this knowledge is crucial to implement meaningful monitoring programs that are able to detect relevant changes in the Sado Estuary, but also in other estuaries with similar hydrodynamic conditions.

## Conclusions

The main objective of the present study was to evaluate how the tide, in its fortnightly and semidiurnal cycles, affect the variability of the water quality parameters in the Sado Estuary. Both factors played a significant role in the variation of physicochemical and biological parameters in the estuary, with the fortnightly tidal cycle showing the most significant influence. Additionally, significant differences were also observed when considering the different sampling stations, revealing clear spatial patterns. Some parameters, such as temperature, SPM and Si(OH)_4_ reached higher values at LW. Therefore, it was possible to conclude that the water derived from Sado River presented higher temperatures than the coastal ocean throughout the sampling period. The implications of the tidal variability in the water quality assessment were also analyzed, under the WFD and, in general, flood seemed to mitigate possible effects of pollution in the water quality of the system. To assess the worst-case scenario in water quality of the Sado Estuary, low water should be chosen as the sampled tidal phase. Lastly, the estuary can be considered a source of nutrients even under meteorological drought conditions.

The present study is a relevant contribution to move towards an effective implementation of water quality monitoring programs and emphasizes the importance in detecting trends only observable in a small-time scale, important for aquaculture purposes and regional satellite remote sensing studies. Also, it will support the implementation of hydrological models, contributing to the development of relevant management tools. This evaluation of the tidal variability influence on the estuary contributes not only to the maintenance of a good water quality conditions, but also to enhance regional sustainable development.

In the future, it would be important to extend this analysis to more areas of the estuary and conduct more campaigns throughout the year, to further discuss these topics. Additionally, more information could be gained by extending the present study to the water column, to evaluate how the tide affects the whole set of the analyzed parameters through the water column.

## Supplementary Information

Supplementary Information.
