# Supplementary material for: Tidal variability of water quality parameters in a mesotidal estuary (Sado Estuary, Portugal)
Source: Sci Rep. 2021 Nov 30;11:23112. doi: 10.1038/s41598-021-02603-6 (PMC8633344; doi:10.1038/s41598-021-02603-6)
Supplement: Supplementary file 1 — Supplementary Information. [file 41598_2021_2603_MOESM1_ESM.docx]

Supplementary Materials

**Table S1** Designation and geographical coordinates of the sampling stations, and their average depth.

| **Station (Stn.)** | **Designation** | **Latitude (º N)** | **Longitude (º W)** | **Mean depth (m)** |
| --- | --- | --- | --- | --- |
| A | Outão | 38.48667 | 008.93000 | 40 |
| B | Setúbal | 38.51809 | 008.89838 | 7 |
| C | Troia | 38.49278 | 008.88167 | 18 |
| D | Lisnave | 38.47083 | 008.79389 | 11 |

**Table S2** Analitycal performance of the reported measurements. The presented ranges refer to the interval of concentrations to which the uncertainty is applicable. LoQ – Limit of Quantification; U and U′ – absolute and relative expanded uncertainty for approximately 95% confidence level using a coverage factor of 2. (*) Relative uncertainties presented in % and absolute uncertainties presented with the reported unit.

| **Parameter** | **LoQ** | **Range** | **Uncertainty, U or U’ (*)** |
| --- | --- | --- | --- |
| T | 0 ºC | 0 – 30 ºC | 0.1 ºC |
| S | 2.000 | 2.000 – 42.000 | 0.009 |
| DO (electrochemical) | 0.12 mg-O2/L | 0.12 – 2.00 mg-O_2_/L | 21.9 % |
|  |  | ≥ 2.00 mg-O_2_/L | 15.4 % |
| DO  (iodometric) |  | 0.12 – 2.00 mg-O_2_/L | 30.8 % |
|  |  | ≥ 2.00 mg-O_2_/L | 7.78 % |
| pH | 2.00 | 2.00 – 10.00 | 0.06 |
| SPM | 2.0 mg/L | 2.00 – 6.00 mg/L | 28.6 % |
|  |  | ≥ 6.00 mg/L | 11.2 % |
| Chl_a | 0.10 µg/L | ≥ 0.10 µg/L | 10.0 % |
| NO_3_^-^ + NO_2_^-^ | 0.50 µmol/L | 0.50 – 1.00 µmol/L | 19.7 % |
|  |  | ≥ 1. 00 µmol/L | 5.77 % |
| NH_4_^+^ | 1.00 µmol/L | 1.00 – 3.00 µmol/L | 32.8 % |
|  |  | ≥ 3. 00 µmol/L | 13.3 % |
| NO_2_^-^ | 0.100 µmol/L | 0.100 – 0.200 µmol/L | 14.1 % |
|  |  | ≥ 0.200 µmol/L | 5.28 % |
| PO_4_^3-^ | 0.200 µmol/L | 0.200 – 0.600 µmol/L | 18.8 % |
|  |  | ≥ 0.600 µmol/L | 8.38 % |
| Si(OH)_4_ | 0.30 µmol/L | 0.30 – 0.90 µmol/L | 24.0 % |
|  |  | ≥ 0.90 µmol/L | 9.86 % |

**Table S3** Information regarding tidal range and mean daily river flow for each sampling campaign.

| Sampling Campaign | Tidal Condition | Tidal Range (m)^1^ | Mean Daily Flow – Sado River (m^3^/s)^2^ |
| --- | --- | --- | --- |
| 2018-05-08 | Neap Tide | 1.4 - 2.6 | 1.76 |
| 2018-05-15 | Spring Tide | 0.4 - 3.6 | 0.70 |
| 2018-11-08 | Spring Tide | 0.5 – 3.5 | 1.11 |
| 2018-11-16 | Neap Tide | 1.4 – 2.6 | 1.03 |
| 2019-06-18 | Spring Tide | 0.7 -3.4 | 0.00 |
| 2019-06-26 | Neap Tide | 1.3 – 2.7 | 0.00 |

^1^ Data from Instituto Hidrográfico (Setúbal-Troia port). For more information see https://www.hidrografico.pt

^2^ Data from Sistema Nacional de Informação de Recursos Hídricos (Moinho da Gamitinha station). For more information see https://snirh.apambiente.pt

**Table S4** PERMANOVA test results. Significant differences (p-values under 0.05) are bold.

| Factors | df | SS | MS | Pseudo-F | p-value (perm) | Unique perms |
| --- | --- | --- | --- | --- | --- | --- |
| Station | 3 | 188.67 | 62.89 | 8.4806 | **0.0001** | 9936 |
| Tidal_phase(Station) | 12 | 73.61 | 6.13 | 1.5191 | **0.0124** | 9852 |
| Tidal_amplitude(Station) | 4 | 95.99 | 24.00 | 5.3832 | **0.0001** | 9923 |
| Month(Station) | 8 | 452.26 | 56.53 | 11.0250 | **0.0001** | 9895 |

| Station Groups | t | p-value (perm) | Unique perms |
| --- | --- | --- | --- |
| D, B | 2.6963 | **0.0022** | 8782 |
| D, A | 5.7820 | **0.0001** | 8808 |
| D, C | 3.6140 | **0.0001** | 8880 |
| B, A | 1.9955 | **0.0236** | 8851 |
| B, C | 1.3401 | 0.0992 | 8820 |
| A, C | 2.0701 | **0.0213** | 8848 |
|  |  |  |  |

**Table S5** Pairwise test evaluating possible significant differences between the sampling stations. Significant differences (p-values under 0.05) are bold.

**Table S6** Eigenvalues, variance contribution, accumulated contribution rate of the principal components analysis (PCA) and factors loadings in principal components, considering the data collected under neap tide.

|  | **Eigenvalues** | | | |  |
| --- | --- | --- | --- | --- | --- |
|  | PC | Eigenvalues | %Variation | Cum.%Variation |  |
|  | 1 | 3.720 | 33.8 | 33.8 |  |
|  | 2 | 2.680 | 24.4 | 58.2 |  |
|  | 3 | 1.860 | 16.9 | 75.1 |  |
|  | 4 | 1.010 | 9.2 | 84.3 |  |
|  | 5 | 0.710 | 6.5 | 90.7 |  |
|  | |  |  |  |  |
| **Eigenvectors** | | | | | |
| **(Coefficients in the linear combinations of variables making up PC's)** | | | | | |
| Parameter | PC1 | PC2 | PC3 | PC4 | PC5 |
| T | -0.201 | -0.086 | -0.613 | 0.191 | -0.129 |
| pH | 0.412 | -0.070 | 0.085 | -0.142 | -0.412 |
| S | -0.404 | -0.299 | 0.243 | 0.118 | -0.061 |
| %DO | 0.370 | -0.100 | 0.190 | 0.227 | 0.562 |
| SPM | -0.351 | -0.083 | -0.363 | 0.363 | 0.114 |
| NO_2_^-^ | -0.271 | 0.430 | 0.263 | -0.086 | 0.235 |
| NO_3_^-^ | 0.404 | 0.144 | -0.364 | -0.023 | -0.217 |
| NH_4_^+^ | 0.057 | 0.256 | 0.299 | 0.665 | -0.470 |
| PO_4_^3-^ | -0.063 | 0.546 | 0.032 | 0.231 | 0.015 |
| Si(OH)_4_ | 0.098 | 0.513 | -0.305 | -0.108 | 0.235 |
| Chl_*a* | 0.335 | -0.221 | -0.074 | 0.481 | 0.320 |

**Table S7** Eigenvalues, variance contribution, accumulated contribution rate of the principal components analysis (PCA) and factors loadings in principal components, considering the data collected under spring tide.

|  | **Eigenvalues** | | | |  |
| --- | --- | --- | --- | --- | --- |
|  | PC | Eigenvalues | %Variation | Cum.%Variation |  |
|  | 1 | 3.840 | 35.0 | 35.0 |  |
|  | 2 | 2.050 | 18.7 | 53.6 |  |
|  | 3 | 1.310 | 11.9 | 65.5 |  |
|  | 4 | 1.080 | 9.8 | 75.3 |  |
|  | 5 | 0.999 | 9.1 | 84.4 |  |
|  | | | | | |
| **Eigenvectors** | | | | | |
| **(Coefficients in the linear combinations of variables making up PC's)** | | | | | |
| Parameter | PC1 | PC2 | PC3 | PC4 | PC5 |
| T | 0.059 | -0.454 | 0.327 | -0.331 | -0.378 |
| pH | 0.103 | 0.300 | -0.070 | -0.170 | -0.802 |
| S | -0.331 | 0.162 | 0.309 | 0.479 | -0.166 |
| %DO | 0.455 | 0.012 | -0.003 | 0.129 | -0.049 |
| SPM | -0.186 | -0.508 | 0.393 | 0.041 | -0.115 |
| NO_3_^-^ | -0.403 | 0.077 | -0.341 | -0.110 | 0.002 |
| NO_2_^-^ | -0.392 | 0.145 | -0.188 | -0.202 | -0.264 |
| NH_4_^+^ | 0.134 | -0.349 | -0.616 | -0.033 | -0.083 |
| PO_4_^3-^ | -0.003 | -0.299 | -0.275 | 0.721 | -0.291 |
| Si(OH)_4_ | -0.316 | -0.413 | -0.172 | -0.187 | 0.100 |
| Chl_*a* | 0.448 | -0.106 | -0.043 | -0.069 | -0.021 |

**Table S8** Pairwise test evaluating possible significant differences between neap (NT) and spring tide (ST) conditions in the different sampling stations. Significant differences (p-values under 0.05) are bold.

| Stations | Groups | t | p-value (perm) | Unique perms |
| --- | --- | --- | --- | --- |
| A | NT, ST | 1.6695 | **0.0384** | 7852 |
| B | NT, ST | 1.9701 | **0.0082** | 7886 |
| C | NT, ST | 1.2986 | 0.1642 | 7908 |
| D | NT, ST | 4.6187 | **0.0001** | 7848 |

**Table S9** Eigenvalues, variance contribution, accumulated contribution rate of the principal components analysis (PCA) and factors loadings in principal components, considering the data collected at station A.

|  | Eigenvalues | | | |  |
| --- | --- | --- | --- | --- | --- |
|  | PC | Eigenvalues | %Variation | Cum.%Variation |  |
|  | 1 | 2.570 | 41.2 | 41.2 |  |
|  | 2 | 1.110 | 17.8 | 59.0 |  |
|  | 3 | 0.999 | 16.0 | 75.0 |  |
|  | 4 | 0.497 | 8.0 | 83.0 |  |
|  | 5 | 0.372 | 6.0 | 88.9 |  |
|  |  |  |  |  |  |
| Eigenvectors  (Coefficients in the linear combinations of variables making up PC's) | | | | | |
| Parameter | PC1 | PC2 | PC3 | PC4 | PC5 |
| T | -0.211 | 0.496 | 0.473 | -0.478 | 0.067 |
| pH | 0.204 | -0.403 | 0.785 | 0.338 | 0.104 |
| S | -0.202 | 0.089 | -0.023 | 0.135 | -0.099 |
| %DO | 0.614 | -0.028 | -0.044 | -0.414 | -0.424 |
| SPM | -0.155 | 0.174 | 0.045 | -0.219 | 0.478 |
| NO_3_^-^ | -0.293 | -0.424 | -0.315 | 0.003 | 0.133 |
| NO_2_^-^ | -0.311 | -0.580 | 0.132 | -0.586 | -0.180 |
| NH_4_^+^ | 0.099 | -0.070 | 0.045 | -0.024 | 0.351 |
| PO_4_^3-^ | -0.064 | 0.015 | -0.073 | 0.059 | 0.092 |
| Si(OH)_4_ | -0.116 | -0.132 | 0.003 | -0.148 | 0.248 |
| Chl_*a* | 0.512 | -0.123 | -0.177 | -0.224 | 0.568 |

**Table S10** Eigenvalues, variance contribution, accumulated contribution rate of the principal components analysis (PCA) and factors loadings in principal components, considering the data collected at station B.

|  | Eigenvalues | | | |  |
| --- | --- | --- | --- | --- | --- |
|  | PC | Eigenvalues | %Variation | Cum.%Variation |  |
|  | 1 | 3.610 | 39.7 | 39.7 |  |
|  | 2 | 1.900 | 20.9 | 60.6 |  |
|  | 3 | 1.580 | 17.3 | 77.9 |  |
|  | 4 | 0.689 | 7.6 | 85.5 |  |
|  | 5 | 0.523 | 5.8 | 91.3 |  |
|  |  |  |  |  |  |
| Eigenvectors  (Coefficients in the linear combinations of variables making up PC's) | | | | | |
| Parameter | PC1 | PC2 | PC3 | PC4 | PC5 |
| T | -0.026 | 0.102 | -0.475 | -0.127 | -0.150 |
| pH | 0.243 | 0.149 | 0.544 | 0.011 | -0.626 |
| S | -0.211 | -0.138 | -0.039 | 0.023 | 0.086 |
| %DO | 0.314 | 0.413 | -0.029 | 0.559 | -0.055 |
| SPM | -0.253 | -0.108 | -0.455 | -0.001 | -0.722 |
| NO_3_^-^ | -0.053 | -0.227 | 0.406 | -0.130 | -0.089 |
| NO_2_^-^ | -0.159 | -0.319 | 0.178 | -0.107 | -0.173 |
| NH_4_^+^ | 0.688 | -0.504 | -0.163 | -0.302 | 0.014 |
| PO_4_^3-^ | 0.281 | -0.396 | -0.144 | 0.611 | -0.060 |
| Si(OH)_4_ | -0.062 | -0.080 | 0.089 | 0.065 | -0.082 |
| Chl_*a* | 0.385 | 0.443 | -0.132 | -0.417 | -0.076 |

**Table S11** Eigenvalues, variance contribution, accumulated contribution rate of the principal components analysis (PCA) and factors loadings in principal components, considering the data collected at station D.

|  | Eigenvalues | | | |  |
| --- | --- | --- | --- | --- | --- |
|  | PC | Eigenvalues | %Variation | Cum.%Variation |  |
|  | 1 | 5.740 | 42.9 | 42.9 |  |
|  | 2 | 3.940 | 29.5 | 72.4 |  |
|  | 3 | 1.370 | 10.3 | 82.6 |  |
|  | 4 | 0.756 | 5.6 | 88.3 |  |
|  | 5 | 0.478 | 3.6 | 91.9 |  |
|  |  |  |  |  |  |
| Eigenvectors  (Coefficients in the linear combinations of variables making up PC's) | | | | | |
| Parameter | PC1 | PC2 | PC3 | PC4 | PC5 |
| T | 0.247 | -0.021 | 0.584 | 0.384 | -0.278 |
| pH | -0.088 | -0.455 | 0.222 | -0.729 | -0.110 |
| S | 0.454 | 0.392 | -0.141 | -0.389 | 0.242 |
| %DO | -0.034 | -0.275 | -0.169 | -0.046 | -0.410 |
| SPM | 0.450 | 0.364 | -0.018 | -0.238 | -0.518 |
| NO_3_^-^ | -0.478 | 0.179 | 0.065 | -0.266 | -0.071 |
| NO_2_^-^ | -0.206 | 0.299 | 0.135 | -0.061 | 0.199 |
| NH_4_^+^ | -0.090 | 0.060 | -0.643 | 0.116 | -0.011 |
| PO_4_^3-^ | -0.195 | 0.321 | -0.059 | -0.070 | -0.437 |
| Si(OH)_4_ | -0.400 | 0.217 | -0.011 | 0.098 | -0.349 |
| Chl_*a* | 0.213 | -0.394 | -0.347 | 0.093 | -0.241 |

**Table S12** Average values observed for each parameter at high (HW) and low water (LW), considering the data obtained during the 6 campaigns under neap and spring tide conditions, for each sampling station. In red are the highest average values obtained for each station. N – number of samples.

| Parameter | A | | B | | C | | D | |
| --- | --- | --- | --- | --- | --- | --- | --- | --- |
|  | HW | LW | HW | LW | HW | LW | HW | LW |
| T | 15.3 | 16.3 | 16.3 | 17.6 | 15.5 | 17.3 | 17.5 | 18.6 |
| pH | 8.1 | 8.0 | 8.0 | 8.0 | 8.1 | 8.0 | 8.0 | 7.9 |
| S | 35.8 | 35.4 | 35.6 | 35.6 | 35.3 | 35.2 | 34.7 | 34.6 |
| %DO | 96.8 | 97.0 | 95.8 | 103.5 | 99.8 | 92.4 | 95.0 | 93.2 |
| SPM | 1.6 | 4.2 | 2.4 | 7.8 | 2.2 | 4.5 | 5.7 | 11.0 |
| NO_2_^-^ | 0.3 | 0.2 | 0.4 | 0.2 | 0.5 | 0.3 | 0.4 | 0.4 |
| NO_3_^-^ | 2.2 | 3.3 | 4.7 | 3.2 | 3.5 | 3.6 | 4.7 | 5.6 |
| NH_4_^+^ | 0.5 | 1.9 | 1.1 | 5.4 | 0.7 | 1.4 | 1.3 | 1.9 |
| PO_4_^3-^ | 0.2 | 0.3 | 0.3 | 0.8 | 0.2 | 0.5 | 0.4 | 0.6 |
| Si(OH)_4_ | 2.5 | 6.8 | 5.6 | 5.3 | 4.8 | 11.8 | 14.4 | 22.4 |
| Chl_*a* | 2.3 | 4.1 | 2.9 | 3.4 | 4.0 | 3.3 | 3.5 | 2.8 |
|  |  |  |  |  |  |  |  |  |
| N | 5 | 5 | 4 | 4 | 6 | 6 | 5 | 5 |
